# The Study of Prescribing Errors Among General Dentists

**DOI:** 10.5539/gjhs.v8n4p32

**Published:** 2015-07-30

**Authors:** Solmaz Araghi, Rohollah Sharifi, Goran Ahmadi, Mahsa Esfehani, Fatemeh Rezaei

**Affiliations:** 1 Endodontics Department, School of Dentistry, Kermanshah University of Medical Sciences, Kermanshah, Iran; 2 School of Dentistry, Kermanshah University of Medical Sciences, Kermanshah, Iran; 3 Oral Medicine Department, School of Dentistry, Qazvin University of Medical Sciences, Qazvin, Iran; 4 Oral Medicine Department, School of Dentistry, Kermanshah University of MedicalSciences, Kermanshah, Iran

**Keywords:** errors in prescribing, medication, general dentists

## Abstract

**Introduction:**

In dentistry, medicine often prescribed to relieve pain and remove infections. Therefore, wrong prescription can lead to a range of problems including lack of pain, antimicrobial treatment failure and the development of resistance to antibiotics.

**Materials and Methods:**

In this cross-sectional study, the aim was to evaluate the common errors in written prescriptions by general dentists in Kermanshah in 2014. Dentists received a questionnaire describing five hypothetical patient and the appropriate prescription for the patient in question was asked. Information about age, gender, work experience and the admission in university was collected. The frequency of errors in prescriptions was determined. Data by SPSS 20 statistical software and using statistical t-test, chi-square and Pearson correlation were analyzed (0.05> P).

**Results:**

A total of 180 dentists (62.6% male and 37.4% female) with a mean age of 8.23 ± 39.199 participated in this study. Prescription errors include the wrong in pharmaceutical form (11%), not having to write therapeutic dose (13%), writing wrong dose (14%), typos (15%), error prescription (23%) and writing wrong number of drugs (24%). The most frequent errors in the administration of antiviral drugs (31%) and later stages of antifungal drugs (30%), analgesics (23%) and antibiotics (16%) was observed. Males dentists compared with females dentists showed more frequent errors (P=0.046). Error frequency among dentists with a long work history (P>0.001) and the acceptance in the university except for the entrance examination (P=0.041) had a statistically significant relationship.

**Conclusion:**

This study showed that the written prescription by general dentists examined contained significant errors and improve prescribing through continuing education of dentists is essential.

## 1. Introduction

Prescribing is one of the fundamental pillars of the treatment process. Prescribe appropriate medication, such as proper diagnosis has considerable importance for treatment. Rational prescribing problems in the healthcare system is a overall problem that in addition to developing countries, in developed countries are also observed (Payne, 2011; Skelly, 2010). One of the most documented patterns of drug administration is prescribe evaluation. A prescription can be used as a measure of the quality of medical education, observe the laws and regulations in the medical community, socio-cultural beliefs and medical status of each country. In some countries to improve rational prescribing and drug utilization research has been done (Desalegn, 2013; Ghimire et al., 2009; Aronson, 2006).

Irrational prescribing can be due to errors such as, required amount of drug, or as errors in writing or Abbreviations or pharmaceutical form, drug dosage, administration method and duration of treatment. These errors can lead to ineffective treatment and dangerous, a long illness or worse, harm to the patient and increases the cost of treatment (Grant et al., 2013; Rothwell et al., 2012; Calligaris et al., 2009).

Dentists, like other health care practitioners should have sufficient knowledge about drugs. Observing the prescription principles according to international law for dentists is required. Although dental prescription generally contain pharmaceutics items are limited to providing short-term drug therapy or specific drugs prescribed for dental surgeries but the evidence suggests that in many countries, dentists often do not enjoy a good medical knowledge for this reason, some mistakes in writing prescription occurred (Guzmán-Álvarez et al., 2012). Goud et al. reported that general dentists prescribed more than required antibiotic for root canal therapy (Goud et al., 2012). Mendonca et al. found that in one-fourth of prescriptions written by dentists, medication names were illegible (Mendoca et al., 2010). The Nezafati et al studied 98.05% of written prescriptions by dentists have errors (Nezafati et al., 2009). Ogunbodede et al findings indicate the presence of different types of error in dentist's prescription in term of dosing, frequency and duration of drug use (Ogunbodede et al., 2005).

Several infectious agents, including various viruses, bacteria, mycobacteria, fungi, etc. can affect the oral cavity. Some of these infectious agents are self-limiting, but others require treatment and if not recognized and untreated can cause irreparable lesions (Lie et al., 2000; Southerland et al., 2005; Palmer et al., 2000). Almost 7% of prescriptions containing antibiotics are prescribed by dentists. Although this amount seems small, but given the number of general dentists, high volume of antibiotics consumed according to their request (Sweeney et al., 2004; Jaunay et al., 2000; Saatchi et al., 2012).

Due to the lack of studies about common errors prescribing dentists in relation to common oral infections in Kermanshah city (Iran) and this study aimed to determine errors in prescribing general dentists in Kermanshah in relation to common oral infections done.

## 2. Materials and Methods

In this descriptive-analytical study general dentist's employing in the city of Kermanshah during 2014 were evaluated using a questionnaire. Employment in general dental practice in private offices or dental clinics of Kermanshah city in 2014 as inclusion criteria and lack of agreement to participate in the study as an exclusion criteria dentist were considered.

List and addresses of all general dentists in Kermanshah city were received from the Deputy of Treatment of Kermanshah University of Medical Sciences. By going to the offices or clinics, and after explaining the objectives of the study and approval by the dentist to participate in the study, the questionnaire was delivered to the dentist in the project (students) completed. The name of the dentist is not mandatory in the questionnaire and therefore don’t require a consent form. Data collection for this study was a questionnaire composed of two parts. The first part contained demographic data of the dentists, including age, gender, work experience and how to enter to the university was collected. The second part, which assessed prescription writing errors, contained five questions with open answers.

In each question, the clinical history of a hypothetical patient was described and then the dentist was asked to similar to a real prescription, write the required medication for the patient. After collecting the completed questionnaires, written prescription of the six types of error (pharmaceutical form, typos, not having to write therapeutic dose, writing wrong dose, a wrong administration order and writing wrong number of drugs) were evaluated and each error rate calculated. The relationship between the errors of gender, work experience and how to enter university and medicines type (antibiotics, antifungals, antivirals, and analgesics) were studied. After collecting the completed questionnaires, the data obtained were entered into SPSS 20 statistical software.

Data were analyzed by SPSS 20 statistical software. To describe the frequency tables, frequency percent, graphs, measures of central (median) and measures of dispersion (standard deviation) was used. Data for T-Test and Chi-square tests were used. In this study, a significant level of 0.05> P was considered.

## 3. Results

In the study 180 general practitioners included 113 men, (62.8%) and 67 women (37.2%) between 24 and 57 years, with an average work experience of 7.5 ± 13.0 years were studied. In terms of how to enter university, 12 (6.7%) complementary, 144 (80%) passed the entrance examination and 24 (13.3%) were hygienist. In Tables 1 to 4 the distribution of errors in the antibiotics, antifungals, antivirals medications and analgesics are shown.

**Table 1:** Descriptive information about the types of errors in the medication group of antibiotics

Variable	Category	Frequency	Frequency %
Pharmaceutical form	Yes	29	16.1
	No	151	83.9
	Total	180	100
Typos	Yes	61	33.9
	No	119	166
	Total	180	100
Not having to write dose	Yes	27	0.15
	No	153	0.85
	Total	180	100
Not having to write therapeutic dose	Yes	36	0.20
	No	144	0.80
	Total	180	100
Using the wrong order	Yes	129	71.7
	No	51	28.3
	Total	180	100
Writing wrong number of drugs	Yes	118	65.6
	No	62	34.4
	Total	1850	100

**Table 2:** Descriptive information about the types of errors in antifungal drug

Variable	Category	Frequency	Frequency %
Pharmaceutical form	Yes	96	53.3
	No	84	46.7
	Total	180	100
Typos	Yes	120	66.7
	No	60	33.3
	Total	180	100
Not having to write dose	Yes	108	0.60
	No	72	0.40
	Total	180	100
Not having to write therapeutic dose	Yes	113	62.8
	No	67	37.2
	Total	1480	100
Using the wrong order	Yes	141	78.3
	No	39	21.7
	Total	180	100
Writing wrong number of drugs	Yes	152	84.4
	No	28	15.6
	Total	180	100

**Table 3:** Descriptive information about the types of errors in drug-resistant virus

Variable	Category	Frequency	Frequency %
Pharmaceutical form	Yes	109	60.6
	No	71	39.4
	Total	80	100
Typos	Yes	96	53.3
	No	84	46.7
	Total	180	100
Not having to write dose	Yes	121	67.2
	No	59	32.8
	Total	180	100
Not having to write therapeutic dose	Yes	135	0.75
	No	45	0.25
	Total	180	100
Using the wrong order	Yes	158	87.8
	No	22	122
	Total	180	100
Writing wrong number of drugs	Yes	159	88.3
	No	21	11.7
	Total	180	100

**Table 4:** Descriptive information about the types of medication errors in group housing

Variable	Category	Frequency	Frequency %
Pharmaceutical form	Yes	49	27.2
	No	131	72.8
	Total	180	100
Typos	Yes	87	48/3
	No	93	51.7
	Total	180	100
Not having to write dose	Yes	67	37.2
	No	113	62.8
	Total	180	100
Not having to write therapeutic dose	Yes	68	37.8
	No	112	62.2
	Total	180	100
Using the wrong order	Yes	147	81.7
	No	33	18.3
	Total	180	100
Writing wrong number of drugs	Yes	154	85.6
	No	26	14.4
	Total	180	100

**Table 5:** Descriptive information about the total number of errors in each drug group

Pharmaceutical group	The minimum number of errors	The maximum number of errors	Mean	Standard deviatio
Antibiotics	0	6	2.22	1.51
Antifungal	0	6	4.06	1.84
Anti-virus	0	6	4.32	1.90
Analgesics	0	6	3.18	1.64
Total	0	24	13.78	4.34

The high mean error related to the antiviral drug group, with an average error for each individual 4.32 and lowest average error related to antibiotics medication group with an average error for each individual 2.22. The mean total errors for each description are 13.78 ± 4.34.

**Figure 1:**
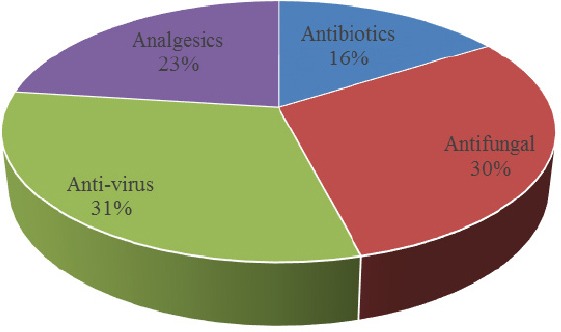
Pharmaceutical Group share of errors recorded in the chart above shows that the largest percentage error is related to antiretroviral therapy.

**Diagram 1:**
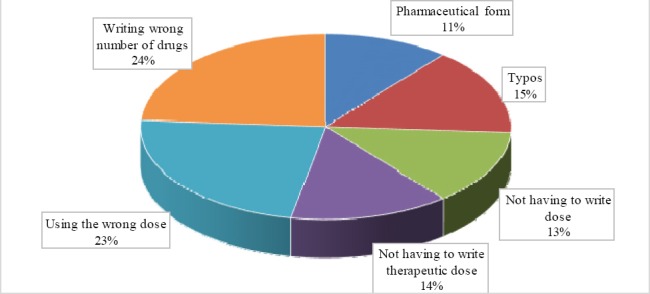
Diagram of the errors listed above each error type shows the highest percentage number medication errors related to incorrect writing number of drugs and the lowest is belong to dosage form

**Figure 2:**
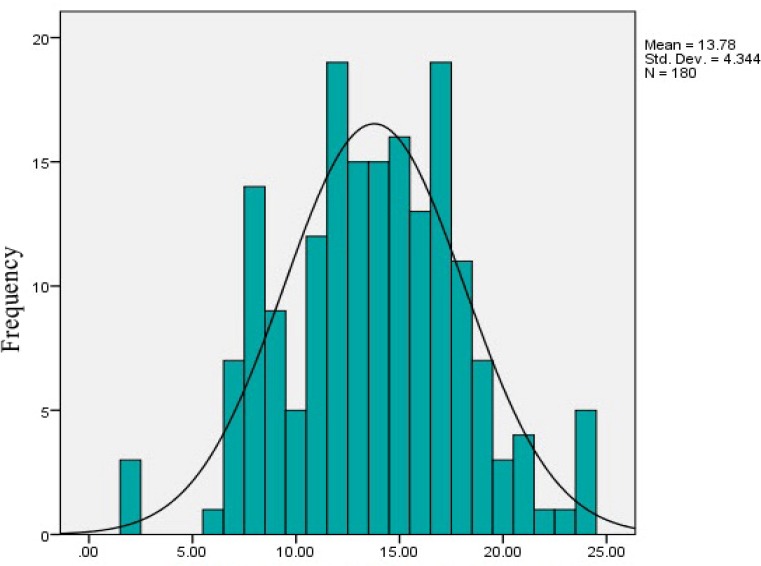
Total number of Prescription errors

**Table 6:** compares the distribution of the errors between women and men

Type o errors	Sex	Pharmaceutical group			

		Antibiotics	Antifungal	Anti-virus	Analgesics
Pharmaceutical form	Woman	11.9%	44.8%	50.7%	16.4%
	Man	18.6%	55.2%	66.4%	33.6%
	P-value	0.241	0.076	0.038	0.012
Typos	Woman	35.8%	70.1%	43.3%	52.2%
	Man	32.7%	64.6%	59.3%	46.%
	P-value	0.673	0.445	0.37	0.419
Not having to write dose	Woman	10.4%	58.2%	58.2%	22.4%
	Man	17.7%	61.1%	72.6%	46.0%
	P-value	0.188	0.706	0.47	0.02
Not having to write therapeutic dose	Woman	10.4%	62.7%	65.7%	25.4%
	Man	25.2%	62.8%	80.5%	45.1%
	P-value	0.014	0.984	0.026	0.008
Using the wrong order	Woman	73.1%	79.1%	88.1%	82.1%
	Man	70.8%	77.9%	87.6%	81.4%
	P-value	0.737	0.847	0.929	0.910
Writing wrong number of drugs	Woman	65.7%	91.0%	85.1%	91.0%
	Man	65.5%	80.5%	90.3%	82.3%
	P-value	0.980	0.060	0.294	0.0\107

**Table 7:** Comparison of Total errors per prescription in both men and women

Variables	Number	Mean	Standard	Results of t-test	

				Test statistic	p-value
Woman	67	12.94	4.33	2.009	0.046
Man	113	14.29	4.30		

The table above descriptive information (frequency percent of errors) to sex shows the result of chi-square tests to compare men and women in terms of the number of errors in each of the drug group. According to the information in the pharmaceutical antibiotic group there was a significant difference between mistakes in writing drugs dosage, in antiviral group between errors in the pharmaceutical form, typos, not having to write dose, and Not having to write therapeutic dose and in analgesics group between pharmaceutical form, not having to write dose, and not having to write therapeutic dose among both men and women (p <0.05). So that all errors in male dentists is significantly more than female dentists.

Based on the results of t-test at a significance level of 05/0 there is a significant difference between average number of errors among two groups of men and women (p=0.046). The mean number of errors is significantly lowers in women than men.

Degree in two categories in the table below is a general category and other categories of complementary product merger of the three groups, and input occupational health, these two categories are based on descriptive information (frequency number errors) the result of the chi-square test to compare the number of errors in each type and class of drug is given Table 8.

**Table 8:** The result of the chi-square test to compare the number of errors in each type and class of drug

Type of error	Academic degree	Pharmaceutical group			

		Antibiotics	Antifungal	Anti-virus	Analgesics
Pharmaceutical form	complementary, occupational health	13.9%	0.75%	77.8%	36.1%
	Overall	16.6%	47.9%	56.3%	0.25%
	P-value	0.685	0.004	0.018	0.180
Typos	complementary, occupational health	36.1%	0.75%	63.9%	58.3%
	Overall	3.33%	64.6%	50.7%	45.8%
	P-value	0.753	0.236	0.156	0.179
Not having to write dose	complementary, occupational health	16.7%	66.7%	80.6%	47.2%
	Overall	14.6%	58.3%	63.9%	34.7%
	P-value	0.754	0.631	0.057	0.165
Not having to write therapeutic dose	complementary, occupational health	16.7%	72.7%	83.3%	44.4%
	Overall	20.8%	60.4%	72.9%	36.1%
	P-value	0.576	0.190	0.197	0.356
Using the wrong order	complementary, occupational health	72.2%	75%	88.9%	88.9%
	Overall	71.5%	79.2%	87.5%	79.9 %
	P-value	0.934	0.587	1.000	0.211
Writing wrong number of drugs	complementary, occupational health	52.8%	88.9%	88.9%	94.4%
	Overall	68.5%	83.3%	88.2%	83.3%
	P-value	0.071	0.411	1.000	0.090

According to the information there is a significant difference between the pharmaceutical antifungal and analgesic medication errors as (p <0.05) in both the error in overall academic degree is significantly lower than other groups.

**Table 9:** Mean total errors per prescription in both academic degree groups

Academic degree	Number	Mean	Standard deviation	Results of t-test	

				Test statistic	p-value
complementary, occupational health	36	15.14	4.21	2.06	0.041
Overall	142	13.48	4.33		

Based on the results of t-test at a significance level of 0.05 there is a significant difference between both groups of academic degree. The mean error for the overall group is significantly lower than other groups.

To investigate the association between the number of errors in each prescription and the work experience of Correlation and Pearson's correlation coefficient was used. The results of this test is positive correlation between the number of errors per prescription and dental history so that with increasing work experience number prescribing errors increased significantly (correlation coefficient = 0.262 and 0.001> p-value).

The scatter plot shows below same thing.

**Diagram 2:**
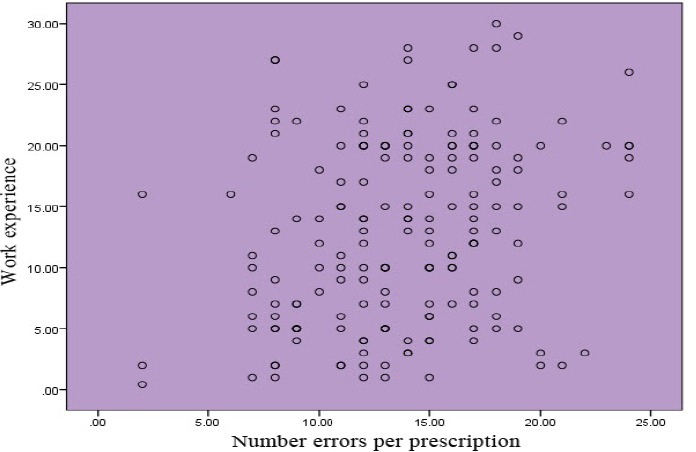
Distribution of Number errors per prescription and work experience

## 4. Discussion

As recommended by the World Health Organization, each prescription should contain information such as the identity of the clinician and the patient, the method of administration, pharmaceutical form, its dosage, frequency, duration of treatment and advice to patients (Moura et al., 2014). Correct prescription is important because the United States alone, approximately 200 thousand people die from drug use of which 100 thousand is due to the excessive use or use of the drug is contraindicated in patients (Gøtzsche et al., 2014).

Because of the importance of correct prescribing, in current study, the frequency of errors in medication commonly examined by general dentists.

In this study, a questionnaire was used to describe the clinical status of five hypothetical patients and the dentist will apply to every case, write the prescription. Previous studies generally prescribing errors in the prescription offered to patients at medical centers were evaluated (Ogunbodede et al., 2005; Sepehri et al., 2001; Palmer et al., 1998). A variety of methods are used to assess prescribing errors. Research in this area may be prospective or retrospective and on the basis of prescribing errors in a range of 0.3% to 39.1% have been reported. Unfortunately, there isn’t a standard method for measuring prescribing errors. Each method has its own advantages and disadvantages and their diversity is very difficult to compare results between studies (Schachter et al., 2009; Dean et al., 2005).

In this study, errors related to prescription drugs, antibiotics, antifungal, antiviral and analgesics were evaluated. For this class of drugs, particularly antibiotics and painkillers are the most common medications prescribed by dentists for patients with dental problems (Jayadev et al., 2014). The findings of the study showed that a wide range of errors in dentist's prescription were considered. Depending on the drug, errors frequency related to the pharmaceutical forms in the 16.1-60.6%, typos 33.9-66.7%, Not having to write therapeutic dose 15-67.2%, wrong in writing the dose 0.20-0.75%, error in the order intake at 71.7-87.8% and errors in writing number of medication 65.6-88.3 percent of cases were reported.

According to the findings of Nezafati et al. in reviewing 666 prescriptions, errors were observed in the name of the medication 94.9%, prescriptions way in the 92.8%, period between doses usage 72.4% and the dose of medicine 60.8% of cases (Nezafati et al., 2009). Similarly, the study of Ogunbodede et al demonstrated that in connection with various drugs, prescription error rate is different. According to their report, the dosages of penicillin, erythromycin, and Ampiclox were correct in all prescriptions. But, the dosage of metronidazole was incorrect in 36% of prescriptions (Ogunbodede et al., 2005).

The findings of the study showed that the maximum error in prescribing antibiotics for order intake (71.7%). In Ogunbodede et al study, in 100% written prescription by dentists; order intake was not observed (Ogunbodede et al., 2005). Most errors in the administration of antifungal drugs (84.4%), antiviral (3/88%), analgesics (85.6%) was related to the number of drug. Al-Khani et al reported the most common error in prescriptions written by doctors at a University Center is wrong dose (54%) (Al-Khani et al., 2014). Guzmán-Álvarez et al survey the dental students, dose-related errors are the most common type of error (74.2%) reported (Guzmán-Álvarez et al., 2012). According to study of Velo, errors in dose selection occur most commonly, and represent >50% of all prescribing faults (Velo et al., 2009).

In the present study, the average error in the questionnaire between 10 and 20 (4.3 ± 13.7) was found. Kia et al examined 850 prescriptions written by dentists found that 97.2% of prescriptions had one or more errors (Kia et al., 2012). Nezafati et al found that 98.05% of prescriptions written by dentists in Tabriz, had errors (Nezafati et al., 2009). Mendonça et al evaluating errors in prescriptions observed that dentists often use acronyms or unconventional in their prescribing in most cases, the order of use, number of medication, dosage, duration of treatment for patients is not authorize or declared (Mendonça et al., 2010). These results indicate a high incidence of errors in prescribing dentist. Some dentists may be due to the time savings, do not pay enough attention to these issues. Such errors may also be due to the lack of systematic information on drugs and limited knowledge of medications.

Several factors can influence prescribing errors. The main reason for prescribing errors is the high number of patients, inadequate knowledge about the inappropriate prescribing and poor pharmacy services have been attributed (Ghoto et al., 2013). Al-Khani et al. identified similarities of the drugs name such as phenytoin and phenobarbital similarities, penicillin, penicillamine, and cyclosporine and cyclophosphamide as main causes of prescribing errors (Al-Khani et al., 2014). According to Moura et al. pharmacology and medication administration training at dental schools have shortcomings and generally many dental students receive instruction, rather than learn systemic administration through the following senior students or teachers (Moura et al., 2014).

Palmer and Martin noted that although prescriptions of general dentists had minor errors and were generally legible, the most common errors related to the duration of required for treatment completion of antibiotics. (Palmer et al., 2000). Errors in prescribing antibiotics are important administration and misuse of antibiotics as the most important cause of antibiotic resistance have been identified. Resistance to antimicrobial agents is an overall health problem due to the overuse of antibiotics worldwide is increasingly higher. This can cause serious infections, complications, prolonged hospital stay and increased morbidity. Excessive prescribing of antibiotics increases the risk of side effects, frequent references and increase drug use and self-limiting infections that are normally associated (Llor et al., 2014; Chate et al., 2006). Concerns about the emergence of bacterial resistance to antibiotics are associated with dental treatment. Amoxicillin, penicillin and metronidazole are the most common antibiotics prescribed by dentists, there is a potential to bacterial resistance and reports on the development of resistance to penicillin in dental infections have been reported (Sweeney et al., 2004; Al-Haroni et al., 2007).

According to the current findings, like the study of Kia et al., there was significant difference between male and female dentists. Some errors (including error in dosage of antibiotics, dosage form, misspelling, and not writing therapeutic dosage for anti-viral agents and error in dosage form and not mentioning the dosage of anti-viral agents) were less frequent in prescriptions of female dentists. However, according to the obtained findings, less frequent errors made by female dentists can be attributed to low workload and higher accuracy mong female dentists (Kia et al., 2013).

How to enter the university has a significant impact on the frequency of errors. So that incidence of the error among dentists are finding their way through National Examination in the country is significantly lower compared to other forms of university entry errors. It was observed that the percentage of errors in prescribing has a significant increase with age. A significant effect of two factors age and the entrance into university on incidence of the error can be explained based on inherent differences or differences in the quality of received education. Obviously, the improvement of dental education in recent years has a significant role on different aspects of young dentist's knowledge such as quality of prescriptions. Based on the literature search, former studies did not review effect of method that dentists entered into dentistry schools and age of dentists on prescribing errors among them.

As a limitation of this study, the applied method does not reflect real prescription practice of dentists. However, this approach has several advantages. Gather information about the quality of prescribing is a simple and low cost method. Furthermore, in this way we can assess the quality of prescribing clinicians in the same condition and compared with each other. As another advantage, this method provides an assessment of medication for a particular situation. Prescriptions collected from hospitals or insurance companies often lack information about the problem for which the drug is administered in such circumstances, even if there was no error in prescriptions incorrectly prescribed medication for the patient. While the method used in this study, the drug is prescribed for a specific clinical condition and authenticity of prescription drugs to the clinical status is evaluable. Furthermore, this method can be prescribing errors before and after learning courses on how Medication review and the effectiveness of such courses in ameliorating prescriptions determined. Similarity in term of the methodology Palmer et al found that the assessment of general practitioners that a significant number of antibiotics inappropriately or incorrectly prescribed dose and duration but after participating in a training program was to improve the quality of prescribing antibiotics (Palmer et al., 2001).

## 5. Conclusion

This study showed that prescribing errors were common among general dentists surveyed. In order to improve the quality of prescriptions written by general dentists training programs to upgrade during college students and conducting training workshops for dentists recommended.
